# Mortality Burden due to Exposure to Outdoor Fine Particulate Matter in Hanoi, Vietnam: Health Impact Assessment

**DOI:** 10.3389/ijph.2022.1604331

**Published:** 2022-04-14

**Authors:** Nguyen T. T. Nhung, Edward Jegasothy, Nguyen T. K. Ngan, Ngo X. Truong, Nguyen T. N. Thanh, Guy B. Marks, Geoffrey G. Morgan

**Affiliations:** ^1^ Biostatistics Department, Hanoi University of Public Health, Hanoi, Vietnam; ^2^ Training and Research Institute for Child Health, Vietnam National Children’s Hospital, Hanoi, Vietnam; ^3^ Sydney School of Public Health and University Centre for Rural Health, Faculty of Medicine and Health, The University of Sydney, Sydney, NSW, Australia; ^4^ Centre for Air Pollution, Energy and Health Research, University of New South Wales, Sydney, NSW, Australia; ^5^ University of Engineering and Technology, Vietnam National University, Hanoi, Vietnam

**Keywords:** Vietnam, mortality burden, PM2.5, HIA, GEMM

## Abstract

**Objective:** This study reports the mortality burden due to PM_2.5_ exposure among adults (age >25) living in Hanoi in 2017.

**Methods:** We applied a health impact assessment methodology with the global exposure mortality model and a PM_2.5_ map with 3 × 3 km resolution derived from multiple data sources.

**Results:** The annual average PM_2.5_ concentration for each grid ranged from 22.1 to 37.2 µg/m³. The district average concentration values ranged from 26.9 to 37.2 µg/m³, which means that none of the 30 districts had annual average values below the Vietnam Ambient National Standard of 25 µg/m^3^. Using the Vietnam Ambient National Standard as the reference standard, we estimated that 2,696 deaths (95% CI: 2,225 to 3,158) per year were attributable to exposure to elevated PM_2.5_ concentrations in Hanoi. Using the Interim Target 4 value of 10 µg/m^3^ as the reference standard, the number of excess deaths attributable to elevated PM_2.5_ exposure was 4,760 (95% CI: 3,958–5,534).

**Conclusion**: A significant proportion of deaths in Hanoi could be avoided by reducing air pollution concentrations to a level consistent with the Vietnam Ambient National Standard.

## Introduction

The negative health effects of particulate matter (PM) with aerodynamic diameter ≤2.5 µm (PM_2.5_), including increased risk of death, have been documented in many previous epidemiologic and toxicological studies. There is consistent evidence of associations between air pollution and risk of death due to circulatory diseases, ischemic heart diseases, chronic obstructive pulmonary diseases, and lung cancer [1, 2]. Furthermore, PM_2.5_ is also linked to disease associated with DNA damage, including cancer [2, 3]. PM_2.5_ contributes to a huge burden of mortality worldwide, estimated at 4.1 million deaths annually in 2019 [4].

The World Health Organization (WHO) has categorized Vietnam among the countries with high concentrations of PM_2.5_. Estimated mean population-weighted PM_2.5_ exposure in Vietnam in 2019 was 20 µg/m^3^ (the range from 16.6 to 25.0 µg/m^3^) [5]. The Government of Vietnam has implemented measures to improve air quality, especially, they established a network of fixed monitoring stations and lower cost sensors. This network provides information for authorities and Hanoi citizens, therefore, is contributing to an increased awareness of air pollution [6]. As a result, annual average PM_2.5_ concentrations in Vietnam have gone down during the period of 2011–2020 [7], though they still exceeded the Vietnamese National Ambient Air Quality Standard (QCVN 05:2013).

Hanoi, the capital, is the most air polluted city in Vietnam. The main emission sources of air pollutants in Hanoi include on-road transport, residential and commercial combustion, especially use of honeycomb coal briquettes, and small industrial activities in craft villages [8].

Several studies have reported short-term effects of PM_2.5_ on children’s respiratory health [9, 10] and on cardiovascular disease in Vietnam [11]. Global studies estimating the health burden attributable to PM_2.5_ exposure for Vietnam have relied on low spatial resolution satellite data covering about 11 × 11 km [5]. Besides, only a recent study reported attributable mortality due to exposure to PM_2.5_ in Ho Chi Minh city in 2017 [12]. However, there has been no estimate based on locally relevant highly resolved data such as district level and/or of the overall health burden attributable to PM_2.5_ exposure using QCVN 05:2013 as a reference. This lack of local data impedes the capacity of local authorities to take action to reduce air pollution and improve health in Vietnam.

The aim of this study was to quantify the annual mortality burden attributable to exposure to high levels of PM_2.5_ air pollution in Hanoi, using highly spatially resolved data acquired locally and with reference to QCVN 05:2013. We also reported the numbers of excess attributable deaths with the reference standards of the Interim Target 4 (IT-4) value and the lowest estimated grid-level value within Hanoi in 2017.

## Methods

### Study Design

This study used a health impact assessment methodology [13, 14] to estimate the mortality burden of ambient PM_2.5_ in Hanoi in 2017. This approach involves the application of established exposure-response functions to the local study population using local mortality data and local measured and modeled exposure data [14]. The mortality burden was estimated as the attributable number of deaths, years of life lost (YLL), and loss of life expectancy (LLE). This study underwent ethical review by the Ethical Committee in Hanoi University of Public Health, decision number 322/2019/YTCC-HĐ3.

### Study Setting

Hanoi is the second largest city in Vietnam covering an area of 3,359 km^2^, just behind Ho Chi Minh city. Hanoi is the capital of Vietnam and had 7.8 million inhabitants in 2017. The city incorporates 30 districts, the population density in the inner district is 4.9 times higher than in outer districts. The districts of Dong Da and Hoan Kiem were the most densely populated with 37,311 and 36,997 persons per km^2^, respectively, (Figure S1, supplementary material). Life expectancy at birth in Hanoi in 2017 was 73.0 for men and 77.9 for women.

### Mortality and Population Size Data

Individual death records including date of death, date of birth or age, address, gender, and causes of death were extracted from the “A6 registration” for Hanoi in 2017 and provided by Hanoi Center of Disease Control. It has good reliability for completeness of death in Vietnam (about 89.3%) and no significant difference in the completeness in the registration between urban and rural regions [15]. The detailed quality assurance process of the A6 death document was published elsewhere [15–17].

Population data by age group and sex for each district in Hanoi in 2017 were provided by the Hanoi Population and Family Planning Agency for each district in Hanoi.

### Ambient PM_2.5_ Concentrations

PM_2.5_ exposure for 2017 in Hanoi was estimated using daily modeled PM_2.5_ concentrations over Vietnam at a 3 × 3 km resolution using a similar modeling approach as previous studies in Europe [18] and the US [19]. Data used in the development of the PM_2.5_ model included ground monitoring stations operated by Vietnam Environment Administration, remote sensing products of aerosol optical depth, meteorological data including temperature, humidity, pressure, wind direction, wind speed, mixing height, and rainfall, and land use data.

We estimated daily PM_2.5_ over Vietnam with a resolution of 3 × 3 km using a mixed effect model (MEM) [18, 19]. The daily MEM was developed from 4-years datasets from 2016–2019 with the following steps: data pre-processing and enhancement, data integration, model development, and daily map estimation.

The pre-processing step converted the ground monitoring station data (multiple stations) into the same format and structure, and outliers were removed using min-max thresholds. All the raster maps were resampled into the same spatial resolution at 3 × 3 km. Because the observations of satellite aerosol optical depth (AOD) are often missing due to cloud coverage, especially in the North of Vietnam, the AOD satellite data were collected from multiple sources and fused together using the Terra regression method to improve the AOD data coverage and quality [20].

After the pre-processing and enhancement step, all data were aligned with daily average PM_2.5_ at the ground from monitoring stations to train the dataset for the daily MEM. In terms of space, different buffers (circular with different diameters) were used to calculate the average value around each station to create new predictor variables. To choose the optimal predictors for the mode, the forward stepwise method [21, 22] and practical experience were applied. The MEM was built on these selected predictors. Since AOD data were often missing due to heavy cloud cover, we proposed to build an auxiliary MEM, which excluded the AOD parameter along with the primary MEM. Based on the two trained MEMs, daily PM_2.5_ maps over Vietnam in 2017 were estimated separately and combined following a principle in which the location without PM_2.5_ estimation from the primary MEM would be supplemented by corresponding PM_2.5_ estimation from the auxiliary model. Thus, the PM_2.5_ maps would have full spatial coverage. Daily PM_2.5_ maps were then converted into monthly and yearly average maps, and then clipped for Hanoi city. Daily 3 × 3 km PM_2.5_ data were summarized to produce annual average maps for the Hanoi study region and for each district. Details of the data sources and model validation are presented in Supplementary material (pages 3–4)**.** We also validated the 2017 annual Vietnam PM_2.5_ map with the annual observations from seven ground stations by matching the pixel value on the map at the ground monitoring station with the annual station’s observation (see Figure S3, supplementary material). The average root mean squared error (RMSE) between annual estimated and observed values in 2017 was 5.82 µg/m³ while the mean relative error (MRE) error was at 22.1%.

### Estimation of Burden of the Premature Death Attributable to Exposure to PM_2.5_

We applied the mortality and PM_2.5_ risk function from the global exposure mortality model (GEMM) [14] for non-communicable diseases (NCD) and lower respiratory infection (LRI) (denoted as GEMM NCD + LRI) to quantify the deaths from non-injury causes that were attributable to exposure to PM_2.5_ among Hanoi residents aged 25 years and older. GEMM used a non-linear exposure-response relationship between ambient PM_2.5_ and mortality (non-accidental) from 41 cohorts from 16 countries including high concentration regions (i.e., China). GEMM provides a hazard ratio for each 5-years age group based on the concentration of PM_2.5_ and the model parameters for the specific age group and outcome. Death at age 25 was not considered in the assessment to avoid the higher proportion of communicable disease mortality [14].

Hazard ratios (HRs) for a given PM_2.5_ concentration were calculated as:

$$GEMM\left(z\right)=ex\mathrm{p\{}\;\theta \;\times \;log\;\left(\frac{z}{\alpha }+1\right)(1+ex\mathrm{p\{}-(z-\frac{\mu }{v}\})\}$$

where z is the difference between PM_2.5_ concentration and the counterfactual concentration, θ, α, μ and v represent model parameters for the shape of the exposure response function. θ and standard error of θ extracted from Burnett [14] are listed in Table S3 for each specific age range.

From the hazard ratio, the burden of mortality relating to exposure to PM_2.5_ for each age group and district was calculated as:

$$AN\;=\left(1-HR\right)\times y_{o}\times pop$$

where AN is the attributable number of premature deaths associated with the concentrations of PM_2.5_ above the counterfactual in each district and age group. HR is the hazard ratio for the specific age group and PM_2.5_ concentration in each district, **y**_**0**:_ is the death rate in the age group and district, and **pop** is the population in each age group and district in Hanoi in 2017.

To limit the error in reporting death registration between districts as indicated in a previous study [15], we applied the death rate for all Hanoi to the district level population by age group to estimate the district level death rate. The attributable number was then summed across age groups to estimate the burden in each district. These numbers were aggregated to calculate the estimate for all of Hanoi.

To assess the burden of PM_2.5,_ we set the counterfactual concentration as defined by QCVN 05:2013 at 25 µg/m^3^. Concentrations in excess of this value, per district, were used in the calculation of attributable deaths.

We also calculated attributable YLL as the sum of the attributable deaths per age group multiplied by the life expectancy in that age group. Life expectancy was estimated using a life table with the baseline mortality rates in the Hanoi population by 5-years age groups. The time of death was assumed to be in the midpoint of each 5-years interval. This method was implemented using the iomlifetR package in R [23]. The LLE, in days, was calculated as the difference between the life expectancy calculated with and without the PM_2.5_ attributable deaths. Confidence intervals (CI) in this study were calculated using the standard errors estimated in the GEMM and only reflected uncertainty in health risk modeling and did not represent the uncertainty in the exposure assessment. The attributable number of each indicator (death or YLL) for each district and all Hanoi was also represented as a rate per 100,000 inhabitants by dividing the attributable number by the population size of the corresponding district.

We also estimated the annual mortality burden due to PM_2.5_ concentration in Hanoi above the lowest estimated grid-level value within Hanoi in 2017 (i.e., 22 µg/m^3^) and IT-4 value (i.e., 10 µg/m^3^).

### Sensitivity Analyses

In addition, we also conducted sensitivity analysis by using log-linear exposure response functions with a uniform effect across age groups. The attributable number was calculated as:

$$RR_{Difference}=e^{\left(\frac{\ln RR}{10}\right)\;X\;\Delta PM_{2.5}}$$

We chose relative risk (RR) estimates for the PM_2.5_ exposure mortality (non-accidental) response function as RR = 1.07 (95% CI:1.04–1.09) of mortality (non-accidental) per 10 µg/m^3^ increase in PM_2.5_ in age groups ≥20 years [24].

The analysis was conducted in R version 4.0.3 (R Core Team Vienna, R Foundation for Statistical Computing, Vienna, Austria) and MS Excel (Microsoft, Redmond, Washington, DC, United States).

## Results

### PM_2.5_ Concentration

**FIGURE 1:**
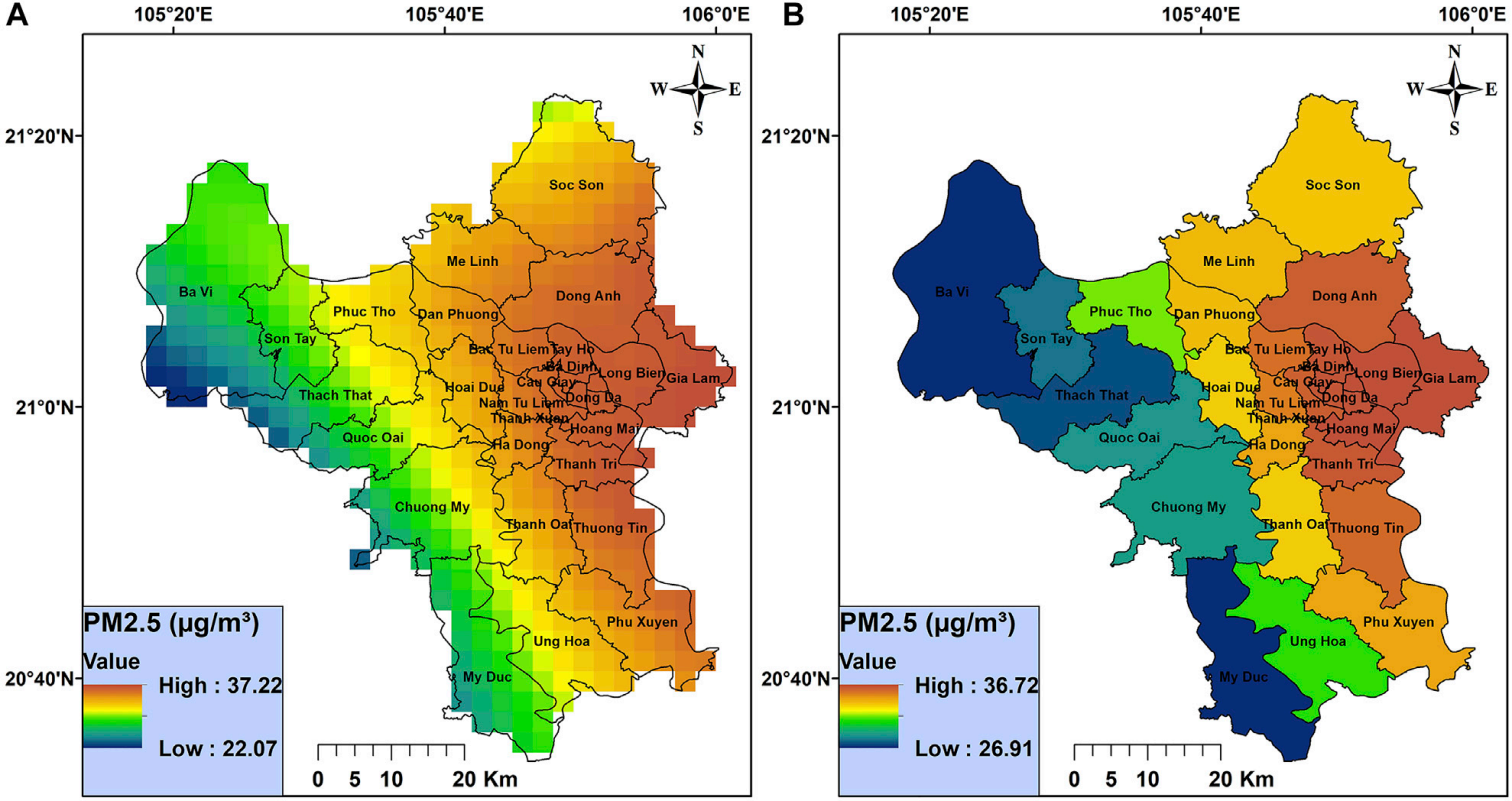
Annual average fine particulate matter concentration map at **(A)** 3 × 3 km resolution and **(B)** district level (Hanoi, Vietnam, 2017).

The 3 × 3 km grid annual average PM_2.5_ concentration map for Hanoi in 2017 is shown in Figure 1A. The annual average PM_2.5_ concentration for each grid ranged from 22.1 to 37.2 µg/m³. The concentrations were higher in the central districts in the city, such as Badinh or Dongda districts. The annual PM_2.5_ concentrations were lower in suburban districts, especially Bavi, Sontay, Thachthat, Quocoai, Chuongmy, and Myduc districts located next to the Bavi mountains.

We aggregated the PM_2.5_ values from the 3 × 3 km grid by district for the purposes of the health impact assessment (Figure 1B). The district average concentration values ranged from 26.9 to 37.2 µg/m³ and all exceeded 25 µg/m³ (QCVN 05:2013).

### Mortality Pattern in Hanoi in 2017

Demographic and death data for Hanoi are presented by district in Table 1. In 2017, Hanoi had 4,892,548 inhabitants aged above 25 representing 62.2% of total inhabitants. Number of deaths due to all causes and excluding injury in 2017 in Hanoi were 27,674 and 26,621, respectively. Death rates were 5.66 per 1,000 all cause deaths and 5.44 per 1,000 deaths without injury (Table 1).

**TABLE 1:** Population, number of deaths, and death rate amongst inhabitants aged above 25 (Hanoi, Vietnam, 2017).

Name of District	Population above 25	Number of deaths		Death rates (per 1,000)	
		All causes	All causes excluding injury	All causes	All causes excluding injury
Total	4,892,548	27,674	26,621	5.66	5.44
Hoan Kiem	131,893	1,178	1,144	8.93	8.67
Ung Hoa	128,070	1,135	1,083	8.86	8.46
Hai Ba Trung	183,815	1,608	1,568	8.75	8.53
Thanh Oai	120,271	922	887	7.67	7.38
Ba Dinh	173,642	1,238	1,182	7.13	6.81
My Duc	139,126	984	938	7.07	6.74
Dan Phuong	103,582	732	704	7.07	6.80
Quoc Oai	117,434	812	777	6.91	6.62
Dong Da	266,727	1,843	1,826	6.91	6.85
Phu Xuyen	131,276	906	872	6.90	6.64
Gia Lam	163,396	1,126	1,086	6.89	6.65
Phuc Tho	120,008	792	751	6.60	6.26
Thuong Tin	156,457	1,017	963	6.50	6.16
Ba Vi	181,748	1,155	1,105	6.35	6.08
Dong Anh	222,838	1,367	1,297	6.13	5.82
Thach That	124,936	764	714	6.12	5.71
Soc Son	190,069	1,126	1,046	5.92	5.50
Chuong My	200,387	1,173	1,103	5.85	5.50
Tay Ho	112,637	625	620	5.55	5.50
Me Linh	134,791	721	683	5.35	5.07
Thanh Tri	151,528	792	771	5.23	5.09
Hoai Duc	165,126	769	744	4.66	4.51
Ha Dong	187,794	795	766	4.23	4.08
Cau Giay	165,222	616	612	3.73	3.70
Long Bien	215,889	779	758	3.61	3.51
Bac Tu Liem	195,065	678	655	3.48	3.36
Son Tay	103,977	340	327	3.27	3.14
Hoang Mai	255,229	824	796	3.23	3.12
Thanh Xuan	190,404	468	462	2.46	2.43
Nam Tu Liem	159,211	389	381	2.44	2.39

This table was sorted into descending values of death rates of all causes.

The highest death rate was observed in Hoan Kiem districts, 8.93 per 1,000 population for all causes amongst inhabitants above 25 years of age. Nam Tu Liem and Thanh Xuan districts had the lowest death rates, 2.46 and 2.44 per 1,000 for death from all causes.

### Attributable Mortality Estimates of Exposure PM_2.5_

The absolute number of deaths, YLL (years), and loss of LE (annual average) attributable to PM_2.5_ exposure are shown in Table 2. The annual mortality attributable to PM_2.5_ in Hanoi above the level of QCVN:05 2013 was 2,696 premature deaths (95% CI: 2,225 to 3,158). The annual YLL and average LE lost due to PM_2.5_ exposure in Hanoi was 68,708 years (95% CI: 56,050 to 81,412) and 659 days (95% CI: 535–785) (∼1.8 years), respectively. We estimated that 9.7% of all deaths amongst the population above 25 years of age in Hanoi were premature deaths attributable to exposure to a PM_2.5_ air pollution level of QCVN 05:2013 (see Table S5, Supplementary material).

**TABLE 2:** The annual burden of mortality relating to exposure to fine particulate matter (Hanoi, Vietnam. 2017).

Name	Attributable Deaths			Years of Life Lost			Loss of Life Expectancy (days)		
	Number	95% CI		Number	95% CI		Number	95% CI	
		Lower	Upper		Lower	Upper		Lower	Upper
All Hanoi	2,696	2,225	3,158	68,708	56,050	81,412	659	535	785
Dong Da	215	177	251	5,164	4,207	6,127	759	616	904
Long Bien	142	118	166	3,628	2,958	4,300	770	625	917
Hoai Duc	135	111	157	3,607	2,944	4,272	768	623	915
Hoang Mai	134	111	157	3,085	2,513	3,661	661	536	787
Dong Anh	133	110	156	3,470	2,831	4,112	749	607	892
Ba Dinh	126	104	148	3,149	2,567	3,734	761	618	907
Thanh Xuan	109	90	127	2,887	2,356	3,420	752	610	896
Gia Lam	104	86	122	2,694	2,197	3,193	792	642	944
Thuong Tin	101	84	118	2,556	2,085	3,030	739	600	880
Ha Dong	97	80	114	2,574	2,101	3,048	692	562	824
Cau Giay	94	78	111	2,487	2,029	2,946	747	606	890
Chuong My	84	69	98	2,194	1,790	2,599	765	621	912
Hoan Kiem	82	68	96	2,181	1,780	2,583	756	613	900
Thanh Tri	80	66	94	2,016	1,643	2,390	758	615	904
Ung Hoa	80	66	93	2,393	1,956	2,829	763	619	910
Soc Son	79	66	93	2,012	1,641	2,385	721	585	859
Tay Ho	79	65	92	2,212	1,807	2,618	731	593	871
Nam Tu Liem	79	65	93	1,964	1,603	2,326	463	377	550
Me Linh	79	65	92	2,181	1,781	2,581	668	542	795
Bac Tu Liem	78	64	91	1,931	1,575	2,289	679	551	808
Hai Ba Trung	75	62	88	1,792	1,461	2,124	558	454	664
Phuc Tho	74	61	87	1,755	1,431	2,080	586	476	697
Thanh Oai	71	59	83	1,760	1,436	2,087	660	536	785
Ba Vi	64	52	74	1,573	1,283	1,864	673	547	802
Son Tay	63	52	74	1,754	1,432	2,076	691	561	823
Dan Phuong	56	46	65	1,251	1,020	1,483	417	339	495
My Duc	51	42	60	1,221	996	1,447	454	369	539
Phu Xuyen	49	40	58	1,174	959	1,391	344	280	408
Quoc Oai	42	35	50	1,011	826	1,197	247	201	293
Thach That	42	35	49	1,032	842	1,221	379	308	450

We used the counterfactual concentration as defined by the Vietnam Ambient Air Pollution Standard (25 µg/m^3^).

Abbreviation: CI: confidence interval.

The mortality burden attributable to PM_2.5_ exposure above QCVN 05:2013 varied by district and was highest in Dong Da, Hoai Duc, and Ba Dinh districts at 57.8 (95% CI: 47.7–67.7), 51.7 (95% CI: 42.6–60.6), and 49.0 (95% CI: 40.5–57.3) per 100,000 population, respectively (see Table S4, Supplementary material). “Green” districts such as Thach That and Ba Vi had the lowest PM_2.5_ attributable death rates of 19.2 (95% CI: 15.8–22.6 per 100,000) and 13.9 (95% CI: 11.4–16.4 per 100,000), respectively.

On the other hand, the numbers of PM_2.5_ attributable deaths due to exposure to PM_2.5_ in Thanh Xuan and Nam Tu Liem were a significant proportion of the total of deaths amongst inhabitants aged above 25 in these districts, 26.6% and 23.5%, respectively (Supplementary material, Table S5). Hai Ba Trung had the lowest contribution, the numbers of PM_2.5_ attributable deaths accounted for 5.6% of the total number of premature deaths amongst inhabitants aged above 25 in the district.

The mortality burden attributable to PM_2.5_ exposure above the “background” level of 22 µg/m^3^ in Hanoi was 3,250 (95% CI: 2,649–3,748) annual premature deaths, or 11.6% of the annual mortality in Hanoi (Table 3, Supplementary Table S5). The mortality burden attributable to PM_2.5_ exposure above the IT-4 of 10 µg/m^3^ was 4,760 (95% CI: 3,958–5,534) annual premature deaths, 17% of the annual deaths in Hanoi.

**TABLE 3:** Number of attributable deaths relating to exposure to fine particulate matter with three scenarios (Hanoi, Vietnam.2017).

	GEMM^1^	WHO^2^ (RR^3^ = 1.07)
	Attributable Deaths (95% CI)	Attributable Deaths (95% CI)
PM_2.5_ lowest levels	3,205 (2,649–3,748)	2,068 (1,220–2,604)
QCVN-2013^4^	2,696 (2,225–3,158)	1,565 (920–1,975)
WHO guideline	4,760 (3,958–5,534)	3,983 (2,388–4,964)

1 Abbreviations: GEMM, global exposure mortality model.

2 WHO, World Health Organization.

3 RR, relative risk.

4 QCVN-2013, the Vietnam Ambient National Standard; CI, confidence interval.

### Sensitivity Analysis

Findings from sensitivity analysis of the 2017 annual PM_2.5_ concentrations compared with the three alternative scenarios (QCVN-2013, IT-4, and the lowest measured value) are presented in Table 3.

## Discussion

We found that the mortality burden due to the annual average of ambient PM_2.5_ above the QCVN 05:2013 PM_2.5_ standard of 25 µg/m^3^ was 2,696 annual deaths (7.3 deaths per day) or 68,708 YLL. The mortality burden from PM_2.5_ exposure above the lower IT-4 of 10 µg/m^3^ was 4,760 annual premature deaths. This suggest that there would be substantial health benefits if the Hanoi government implemented policies that were effective in reducing air pollution to the levels recommended by these standards.

Air quality varied substantially in Hanoi over the period 2010 to 2018, with annual average PM_2.5_ concentrations varying from 29 to 55 µg/m^3^ [25], with an annual average PM_2.5_ concentration in 2017 of 26.9 to 37.2 µg/m^3^, which was in the middle of this range. Our results also illustrate the high concentration of PM_2.5_ in the industrial districts in Hanoi such as Gia Lam and Long Bien, with many small and middle-sized factories located in these districts. In recent years, the Hanoi government has attempted to relocate small factories from urban to suburban areas and moved big factories to other provinces outside Hanoi. This has contributed to recent declines in annual ambient PM_2.5_ concentrations in Hanoi [6, 25].

Population variation between districts is another important factor in the variation in the attributable rate due to exposure to PM_2.5_ between districts. For instance, districts such as Son Tay, Tay Ho, and Dan Phuong have relatively small populations. Hence the overall health burden from PM_2.5_ exposures was low compared to higher population districts. In contrast, Hoan Kiem and Ba Dinh are high-population districts and their associated mortality burdens were the highest, even though the exposure was low compared to many other districts.

Our estimate that mortality attributable to long-term exposure to PM_2.5_ was 34.3 per 100,000 population was slightly lower than the GBOD estimation for all of Vietnam (i.e., 36.82 per 100,000 population in 2017) [4]. That number is much higher that the estimate for metropolitan Bangkok, about 21 per 100,000 population [26]. Metropolitan Bangkok, close to Vietnam, shares similar emission sources, population, and weather, therefore, this confirms the size of the problem in Hanoi. Solving this problem for Hanoi requires attention to sources. Previous studies on sources of air pollution in Hanoi have shown that traffic [27], industrial processes, rice stubble burning, and open burning of waste [28] are major contributors. However, a detailed assessment of the contribution of the various emission sources of Hanoi air pollution is absent to date. More detailed data are required on the relative importance of the various emission sources.

A strength of our study is the use of a high resolution PM_2.5_ concentration map (3 × 3 km) to obtain district-specific PM_2.5_ concentrations. We also used detailed local data on mortality, not previously used in health impact assessment studies in Vietnam. However, the study has several limitations. First, our study estimated district level mortality by applying Hanoi-wide death rates to the local district population. This value was estimated from our data as 5.66‰ for all causes. This is similar to the all-cause death rate for all ages in Hanoi in 2017 [29]. The A6 document that was the source of the deaths data is known to only cover around 86% of the total deaths in Vietnam [15]. Moreover, the A6 document deaths data we used only records the death of inhabitants with permanent addresses in Hanoi. In fact, a large number of inhabitants live in Hanoi without having a permanent address, and death records of them have been issued by their home provinces. So, our estimation of the death rates due to district pollution is likely to be an underestimate, and this would also result in an underestimate for our mortality burden calculations. Second, the GEMM exposure–response function for PM_2.5_ applied in this health impact assessment relies on the results of epidemiology research from other countries and may not capture the true PM_2.5_ and mortality exposure-response relation in Hanoi. Third, the areas of districts in Hanoi differ from that of the grids and this may introduce some bias in assigning concentrations from grid levels to district levels since areas of districts do not align exactly with the grids. However, the concentrations for each district were assigned by using values of grids covering the majority of district areas. Hence, we consider it unlikely that this would have a substantial impact on our results.

### Conclusion

The attributable deaths due to exposure to PM_2.5_ in Hanoi above the Vietnam QCVN 05:2013 of 25 µg/m^3^ was 2,696 deaths or 34.3 per 100,000 inhabitants, with large variation in the mortality burden by district. This means that there would be substantial health benefits by reducing air pollution in Hanoi. While outside the scope of our current study, health risk assessment research can provide estimates of the financial benefits to health from reducing air pollution that can be used in cost-effectiveness assessments of air pollution control measures.
